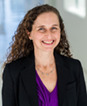# BioTM Buzz (Volume 5, Issue 3): The Future is Bright

**DOI:** 10.1002/btm2.10185

**Published:** 2020-09-16

**Authors:** Samir Mitragotri, Aaron C. Anselmo

**Affiliations:** ^1^ John A Paulson School of Engineering and Applied Sciences Harvard University, Wyss Institute of Biologically Inspired Engineering Cambridge Massachusetts USA; ^2^ Division of Pharmacoengineering and Molecular Pharmaceutics Eshelman School of Pharmacy, University of North Carolina at Chapel Hill Chapel Hill North Carolina USA

No one can predict the future, but that is not going to stop us from taking a shot at it. At BioTM, we do wonder what the future holds for our key scientific areas, including drug delivery, tissue engineering, cell therapy, and biosensors, among many others. Ultimately, the future will become what the future scientists aspire to do. With this realization, we figured that our best shot at predicting the future is by featuring the science from the labs of the scientists of the future. This “Futures” issue features articles from nine researchers who are in the early stages of their independent careers. The Futures issue is a part of AIChE's initiative to feature the emerging leaders in the field. We asked our Editorial Advisory Board to submit nominations for this recognition. Thanks to our strong and supportive Board, we received an overwhelming number of nominations, and we were able to include only a small fraction of the nominations in this inaugural Futures issue. We did not ask the nominees to predict the future but simply invited them to submit a manuscript covering the latest and most exciting research in their lab. Our goal is to feature these talented individuals and bring to you the topics that are on their mind these days. The topics covered in the issue include nanoparticle drug delivery, portable biosensors, tissue engineering, and optimization of drug properties.

## JAMES DAHLMAN

Dr. James Dahlman is an Assistant Professor in the Georgia Tech Wallace H. Coulter Department of Biomedical Engineering. The Lab for Precision Therapies at Georgia Tech, also called the “Dahlman Lab”, works at the interface of drug delivery, nanotechnology, genomics, and gene editing. The Dahlman Lab has developed new methods to quantify nanoparticle pharmacokinetics for up to hundreds of unique nanoparticles in single experiments and leverages this approach to optimize in vivo delivery of nanoparticle therapies for RNA therapy. Dr. Dahlman received his PhD from MIT and Harvard Medical School in 2014, where he studied RNA delivery with Dr. Robert Langer and Dr. Daniel Anderson. Dr. Dahlman then studied RNA design and gene editing as a post‐doc with Dr. Feng Zhang at the Broad Institute. Dr. Dahlman has won the NSF, NDSEG, NIH OxCam, Whitaker Graduate, and LSRF Fellowships; the Weintraub Graduate Thesis Award; the BMES Rita Schaffer Award; the ASGCT Young Investigator Award; and other young investigator awards from Bayer, Controlled Release Society, and others. In this *Futures* issue of *Bioengineering & Translational Medicine*, Dr. Dahlman shares his lab's research on identifying a novel lipid nanoparticle formulation that can deliver mRNA to immune cells, expanding their potential applications beyond hepatocytes.^1^

DOI: 10.1002/btm2.10161 
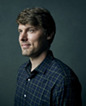


## RIZIA BARDHAN

Dr. Rizia Bardhan is an Associate Professor in the Department of Chemical & Biological Engineering at Iowa State University. Dr. Bardhan's research is focused on the use of both soft and hard nanoparticles for molecular imaging and image‐guided treatment that include immunotherapies, drug delivery, and light‐based therapies. Dr. Bardhan is also an expert in Raman spectroscopy and its applications in metabolic response to treatment in cancer, neurodegenerative diseases, and preterm labor in pregnancy. Dr. Bardhan received her BA in Chemistry and Mathematics from Westminster College, Fulton, MO, and her PhD at Rice University under the supervision of Prof. Naomi Halas and completed her postdoctoral work in the Molecular Foundry at Lawrence Berkeley National Laboratory. Dr. Bardhan is a recipient of the CDMRP Career Development Award and CDMRP Idea Award and was selected for Forbes “Top 30 under 30” Rising Stars of Science and Innovation, among other awards and honors. She was also recognized as one of “40 Women Honorees in 40 Years” by her alma mater, Westminster College. In this *Futures* issue of *Bioengineering & Translational Medicine*, Dr. Bardhan shares her lab's development of a portable biodiagnostic sensor, capable of detecting biomarkers for myocardial infarction in low‐volume samples.^2^

DOI: 10.1002/btm2.10165 
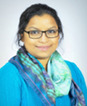


## CÉSAR DE LA FUENTE

Dr. César de la Fuente is the Presidential Assistant Professor at the University of Pennsylvania in the Department of Bioengineering at the University of Pennsylvania. Dr. de la Fuente's laboratory aims to develop the computational tools to expand the antibiotic arsenal, engineer the microbiome, and study and control brain function and behavior. Dr. de la Fuente seeks to develop novel classes of antibiotics using computers. Dr. de la Fuente received his B.Sc. and M.Sc. from the University of Leon and his PhD from the University of British Columbia before completing his postdoctoral work at MIT. He is the recipient of the Langer Prize, ACS Kavli Emerging Leader in Chemistry award, MIT Technology Review Top 35 Innovators Under 35, and ACS Infectious Diseases Young Investigator Award, among other awards. In this *Futures* issue of *Bioengineering & Translational Medicine*, Dr. de la Fuente shares his lab's work toward optimizing the physicochemical properties of wasp venom‐derived antimicrobial peptide polybia‐CP for antiplasmodial and anticancer applications.^3^

DOI: 10.1002/btm2.10167 
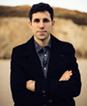


## STEVEN JAY

Dr. Steven Jay is an Associate Professor in the Fischell Department of Bioengineering at the University of Maryland. Dr. Jay's laboratory aims to develop new biotechnologies to address a wide variety of clinical needs while focusing on improving the mechanistic understanding of biotherapeutics to inform rational design of next‐generation approaches. Dr. Jay received his BSBE in Biological Engineering from the University of Georgia and his PhD in Biomedical Engineering from Yale University before completing his postdoctoral work at Brigham and Women's Hospital and Harvard Medical School. Dr. Jay has been the recipient of many research awards, including a NSF CAREER Award, a Young Innovator in Cellular and Molecular Bioengineering Award, and a NIH K99/R00 Pathway to Independence Award. He has also been recognized with a Graduate Mentor of the Year award from the University of Maryland. In this *Futures* issue of *Bioengineering & Translational Medicine*, Dr. Jay shares his lab's insight on the therapeutic potential of extracellular vesicles as carriers for long noncoding RNAs.^4^

DOI: 10.1002/btm2.10172 
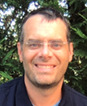


## EUN JI CHUNG

Dr. Eun Ji Chung is an Assistant Professor in the Department of Biomedical Engineering at the University of Southern California and the Dr. Karl Jacob Jr. and Karl Jacob III Early Career Chair. She also has courtesy appointments in Chemical Engineering, Medicine (Nephrology and Hypertension), and Surgery (Vascular Surgery and Endovascular Therapy). Her laboratory is interested in harnessing molecular design and self‐assembly to develop nano‐ to macroscale biomaterials that can be utilized in medicine. Dr. Chung received her BA with honors in Molecular Biology from Scripps College, her PhD from the Department of Biomedical Engineering from Northwestern University, and her postdoctoral training from the Pritzker School of Molecular Engineering at the University of Chicago. Dr. Chung is a recipient of the NIH K99/R00 Pathway to Independence Award and the NIH Director's New Innovator Award and was named 35 Under 35 from the American Institute of Chemical Engineers (AIChE), New Innovator from IEEE‐Nanomed, and Rising Star in Cellular and Molecular Bioengineering from BMES, among others. In this *Futures* issue of *Bioengineering & Translational Medicine*, Dr. Chung shares her lab's progress on optimizing kidney‐targeting nanoparticles for their eventual use in treating chronic kidney diseases.^5^

DOI: 10.1002/btm2.10173 
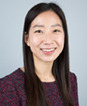


## ELIZABETH NANCE

Dr. Elizabeth Nance is the Clare Boothe Luce Assistant Professor of Chemical Engineering at the University of Washington. Dr. Nance's laboratory integrates in vitro to in vivo models with imaging, molecular biology, and data science tools to extract statistically relevant information that captures changes in the brain that might influence how a therapeutic behaves. The lab uses the information gathered from the application of these tools to design nano‐based therapeutics that can achieve region‐ and cell‐specific targeting in the brain for improved neuroprotection in a variety of brain disease models. Dr. Nance received her BS in Chemical Engineering from North Carolina State University, her PhD in Chemical and Biomolecular Engineering from Johns Hopkins University, and completed her postdoctoral fellowship at the medical school at Johns Hopkins University. Dr. Nance is the recipient of the Presidential Early Career Achievement in Science & Engineering award, the European Union Horizons 2020 Inspiring Young Scientist in Nanomedicine award, 45 Under 45 Young Innovator in Nanobiotechnology award, and the Burroughs Wellcome Fund CASI Award, among many others. In this *Futures* issue of *Bioengineering & Translational Medicine*, Dr. Nance shares her lab's work on modeling ischemic injury to describe how injury and nanoparticle material identity affect interactions with disease‐mediating cells in the brain.^6^

DOI: 10.1002/btm2.10175 
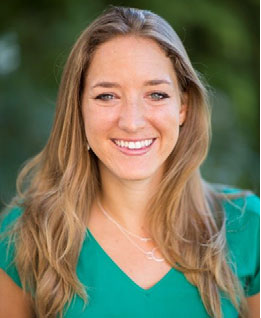


## AMIR SHEIKHI

Dr. Amir Sheikhi is an Assistant Professor of Chemical Engineering and, by courtesy, Biomedical Engineering at The Pennsylvania State University. Dr. Sheikhi's laboratory (Bio‐Soft Materials Lab, B‐SMaL) focuses on developing micro‐ and nanoscale soft material technologies with tailored structure‐property relationships based on natural or seminatural biomaterials for healthcare and environmental applications. Dr. Sheikhi received his BS and MS in Chemical Engineering from the University of Tehran and his PhD in Chemical Engineering from McGill University prior to completing his postdoc at McGill, Harvard and MIT, and then UCLA. Dr. Sheikhi is the recipient of numerous awards and recognitions, including Iranian American Medical Association (IAMA) of Massachusetts Young Investigator Award, selection as a UNIFOR Global Research Fellow, Canadian Institutes of Health Research (CIHR) Postdoctoral Fellowship, The Marcus Wallenberg Delegate Award, and AIChE's 35 Under 35 Award. Dr. Sheikhi's research has been featured in more than 55 publications, 25 seminars, and 9 reports of invention/patent applications. In this *Futures* issue of *Bioengineering & Translational Medicine*, Dr. Sheikhi shares his lab's work on developing in situ forming microporous hydrogels made up of thermostable, photo‐annealable gelatin methacryloyl microgels, a step forward in leveraging gelatin‐based hydrogels for in situ tissue repair at physiological temperature well above the gelatin melting point.^7^

DOI: 10.1002/btm2.10180 
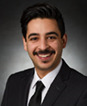


## MARK TIBBITT

Dr. Mark Tibbitt is an Assistant Professor of Macromolecular Engineering in the Department of Mechanical and Process Engineering at ETH Zürich. Dr. Tibbitt's laboratory focuses on combining polymer engineering, synthetic chemistry, and mechanical and bioengineering to design new materials for biomedical applications. Specific applications of the lab focus on engineering hydrogels as extracellular matrix mimics for 3D cell culture, injectable drug delivery systems, the fundamentals of reversible network design, and additive manufacturing of precision biomaterials. Dr. Tibbitt received his BA in Integrated Science and Mathematics from Northwestern University, his PhD in Chemical and Biological Engineering from the University of Colorado Boulder, and completed his postdoctoral studies at MIT as a National Institutes of Health NHLBI postdoctoral fellow. Dr. Tibbitt is the recipient of the Golden Owl for Excellent Teaching from ETH Zürich, a Ruth L. Kirschstein Postdoctoral Fellowship from the NIH, and the Outstanding Biomaterials Thesis Award from the Max Bergmann Center of Biomaterials Dresden. In this *Futures* issue of *Bioengineering & Translational Medicine*, Dr. Tibbitt shares his lab's work to develop screening methods for engineering 3D hydrogels that facilitate myotube formation from primary myoblasts toward skeletal muscle tissue engineering.^8^

DOI: 10.1002/btm2.10181 
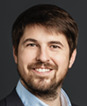


## HADLEY SIKES

Dr. Hadley Sikes is an Associate Professor and the Esther and Harold E. Edgerton Career Development Professor in the Department of Chemical Engineering at MIT. Dr. Sikes's laboratory focuses on engineering biomolecular systems to detect and treat disease in new ways. The lab uses a variety of synthetic and analytical techniques to manipulate and measure the kinetics of biochemical reactions, with an interest in understanding nonlinear reaction networks, as well as the interplay between transport and reaction rates. Dr. Sikes received her BS in Chemistry from Tulane University, her PhD in Physical Chemistry from Stanford University, and completed postdoctoral studies at both the University of Colorado Boulder and Caltech. Dr. Sikes was honored as an innovative young engineer by the National Academy of Engineering as part of the Frontiers of Engineering Symposium and received a Best of BIOT Award from the American Chemical Society and the C2C Award for Graduate Advising at MIT. In this *Futures* issue of *Bioengineering & Translational Medicine*, Dr. Sikes shares her lab's work toward a quantitative and compartment‐specific understanding of redox metabolism in human cells, which is relevant to many disease states, as well as responses or resistance to therapies.^9^

DOI: 10.1002/btm2.10184